# The Immunogenicity of Fractional Intradermal Doses of the Inactivated Poliovirus Vaccine Is Associated With the Size of the Intradermal Fluid Bleb

**DOI:** 10.1093/cid/cix381

**Published:** 2017-04-21

**Authors:** Jack Bibby, Yauba Saidu, Ama Umesi, Ngozi Moneke-Anyanwoke, Adedapo O Bashorun, Mariama Badjie Hydara, Ikechukwu Adigweme, Jane U Adetifa, Michael Okoye, Elishia Roberts, Ralf Clemens, Ananda S Bandyopadhyay, Abdul K Muhammad, Sarah Mulwa, Michael Royals, Courtney Jarrahian, David Jeffries, Beate Kampmann, Ed Clarke

**Affiliations:** 1 Vaccines and Immunity Theme, MRC Unit The Gambia, Fajara;; 2 Research in Infectious Diseases, Rio de Janeiro, Brazil;; 3 Bill & Melinda Gates Foundation, Seattle, Washington;; 4 Thrivant Health, Portland, Maine;; 5 PATH, Seattle, Washington; and; 6 Imperial College London, United Kingdom

**Keywords:** polio, inactivated poliovirus vaccine, intradermal

## Abstract

The immunogenicity of fractional (one-fifth, 0.1 mL) intradermal doses of the inactivated poliovirus vaccine (ID fIPV) is positively correlated with the size of the intradermal fluid bleb. Training of vaccinators for campaign and routine ID fIPV administration should focus on generating an 8- to 10-mm bleb with each injection.

**Clinical Trials Registration:**

NCT01847872

The global effort to eradicate polio is now in its final endgame. Wild-type poliovirus type 2 was declared eradicated in September 2015, and in April 2016 a synchronous switch from the use of the trivalent oral poliovirus vaccine (OPV) containing the type 1, 2, and 3 virus, to the bivalent format containing only the type 1 and 3 virus, occurred globally [1]. The switch was to have been accompanied by the introduction of a routine dose of the (trivalent) inactivated poliovirus vaccine (IPV) in all countries using OPV only to mitigate against the risk of future type 2 disease in the event of a vaccine-derived poliovirus type 2 (VDPV2) outbreak; also, to enhance immunity against poliovirus types 1 and 3 [1]. However, requirements for IPV have outstripped manufacturing capacity and resulted in a vaccine shortage that has both delayed vaccine introduction and limited the vaccine stockpiles available for outbreak response. Consequently, in June 2016, following the detection of a VDPV2 during routine environmental surveillance in Telangana state in India, a campaign using fractional (one-fifth, 0.1 mL) doses of IPV (fIPV) delivered by the intradermal (ID) route was undertaken and included a total of 311 064 children [2]. Certain Indian states and Sri Lanka have also introduced ID fIPV into their routine immunization schedules and, in response to the shortage, the World Health Organization (WHO) has now strongly recommended that ID fIPV be used universally, both in campaigns and routinely, advice which is expected to last until at least mid-2018 [3–5].

Whereas previous studies have confirmed the immunogenicity of ID fIPV, the per-dose seroconversion rates and geometric mean neutralizing antibody titers are consistently lower than those generated by a full (0.5 mL) intramuscular (IM) dose of the same vaccine (Supplementary Table 1). This nested study provides definitive data on the effect of ID fluid bleb size on the immunogenicity of ID fIPV and should be used as a basis for current training and vaccination practice.

## METHODS

### Study Design

The study was nested in a clinical trial undertaken to examine the immunogenicity of ID fIPV and the use of disposable syringe jet injectors (DSJIs) to deliver IPV in 9- and 10-month-old infants in The Gambia (NCT01847872) [6]. All infants had received at least 3 previous doses of the trivalent oral poliovirus vaccine (tOPV) at least 28 days prior to enrollment (see Supplementary Table 2 for full eligibility criteria). The study was approved by The Gambia Government/MRC Joint Ethics Committee and was conducted according to the International Conference on Harmonization Good Clinical Practice and local ethical and regulatory guidelines. Full details of the enrollment procedures have previously been reported [6]. In brief, following informed consent, infants were block-randomized into 1 of 8 groups, of which 4 provided data for this study. Two groups received ID fIPV, the first using a needle and syringe (N&S) and the second using a DSJI (Tropis, PharmaJet). Two groups received a full IM dose of IPV using either a N&S or a DSJI (Stratis, PharmaJet). Immediately following each injection, any fluid lost onto the skin was absorbed with a Whatman filter paper disc and the ID bleb size was measured with a clear plastic ruler. The area of wetness on the filter paper was outlined and later compared in an observer-blinded fashion to a standard scale, allowing the volume of fluid loss to be quantified.

### Serological Endpoint Measurement

A prevaccination serum sample was taken prior to IPV administration, and a postvaccination sample was taken 4–6 weeks later. There were no systematic differences in the timing of the postvaccination sample between groups. Poliovirus serotypes 1, 2, and 3 neutralizing antibody titers were assessed in a laboratory observer–blinded fashion, as previously reported [7]. Serial 2-fold dilutions from a starting dilution of 1 in 8 were undertaken until an endpoint titer was obtained. Seropositivity was defined as reciprocal neutralizing antibody titers of ≥8. The rate of seroconversion (seronegative prevaccination to seropositive postvaccination) and the number of infants who were seropositive at baseline and who had a 4-fold rise in antibody titers were calculated. These 2 groups were combined to provide a measure of the total response to the vaccine.

### Statistical Analysis

Summary statistics were generated and the relationship between ID bleb size and cutaneous fluid loss and immunogenicity was assessed using Spearman correlation coefficient. The 95% confidence intervals (CIs) of the correlation coefficient were derived using the Fisher *r*-to-*z* transformation [8]. All analyses were done using Stata software (release 12.1).

## RESULTS

### Study Population, Baseline Seroprevalence, and Seroresponse

Seven hundred 9- and 10-month-old infants were recruited and randomized into 1 of the 4 groups and provided data for analysis in this study. The infants had received between 4 and 7 (mean, 4.9) doses of tOPV prior to the current study—the last dose being at least 4 weeks prior to enrollment. Of the 700 infants, 177 each received ID fIPV using a N&S and a DSJI. One hundred seventy-eight received a full IM dose of IPV using a N&S and 168 infants received a full IM dose using a DSJI.

The baseline poliovirus neutralizing antibody titer seropositivity rate was 85.9%–89.9% for poliovirus type 1, 96.1%–98.9% for poliovirus type 2, and 74.0%–82.7% for poliovirus type 3. Overall, 66.9% and 66.1% of infants had a poliovirus type 1 immune response following IPV administration by the IM N&S and IM DSJI, respectively, compared to 55.9% and 43.5% for the same ID fIPV administration methods. The equivalent figures for poliovirus type 2 were 72.5% and 59.5% for the IM routes compared with 58.2% and 41.8% by the ID routes, and for poliovirus type 3, 82.6% and 81.0% by the IM routes compared with 80.2% and 69.5% by the ID routes (Supplementary Table 3).

### Effects of Bleb Size and Vaccine Loss on Intradermal Doses of the Inactivated Poliovirus Vaccine Immunogenicity

The median bleb size generated by the ID N&S was 8 mm with an interquartile range (IQR) of 7–8 mm, which was significantly larger (*P* < .0001) than the median size of 6 mm and interquartile range of 5–7 mm generated by the ID DSJI (Table 1). There was a significant positive correlation between bleb size and the total immune response for all 3 poliovirus serotypes when combining data from the ID N&S and ID DSJI. The correlation remained for all 3 serotypes when examining administration by the ID DSJI and for serotype 1 when examining N&S-based administration (Table 1). A positive correlation was also noted when the relationship between bleb size and postvaccination antibody titers was examined (Supplementary Table 4). Furthermore, the immune responses to ID fIPV in infants with an 8- to 10-mm fluid bleb were comparable to those generated by a full IM dose of the same vaccine. Thus, 65.8% (95% CI, 57.1%–74.5%; 75/114) of infants with a bleb size of 8–10 mm seroconverted or had a 4-fold rise in antibody titers to serotype 1 compared with 66.5% (95% CI, 61.5%–71.4%; 230/346) of those who received a full IM dose of the vaccine. The equivalent figures for serotype 2 were 58.8% (95% CI, 49.7%–67.8%; 67/114) by the ID route compared with 66.2% (95% CI, 61.2%–71.2%; 229/346) by the IM route and for serotype 3, 86.0% (95% CI, 79.6%–92.3%; 98/114) by the ID route compared with 81.8% (95% CI, 77.7%–85.9%; 283/346) by the IM route (Table 1 and Supplementary Table 3).

**Table 1.** Intradermal Fluid Bleb Measurements and Total Immune Responses to Fractional Intradermal Doses of Inactivated Polio Vaccine

		Distribution of ID Bleb Sizes		Serotype 1			Serotype 2			Serotype 3		
				Total Response (Seroconversion + 4-Fold Titer Rise)			Total Response (Seroconversion + 4-Fold Titer Rise)			Total Response (Seroconversion + 4-Fold Titer Rise)		
		no./No. (%)		no./No.; % (95% CI)			no./No.; % (95% CI)			no./No.; % (95% CI)		
		ID N&S	ID DSJI	ID N&S	ID DSJI	Combined	ID N&S	ID DSJI	Combined	ID N&S	ID DSJI	Combined
All data		177	177	99/177; 55.9% (48.3–63.4)	77/177; 43.5% (36.1–51.1)	176/354; 49.7% (44.4–55.1)	103/177; 58.2% (50.6–65.5)	74/177; 41.8% (34.5–49.4)	177/354; 50.0% (44.7–55.3)	142/177; 80.2% (73.6–85.8)	123/177; 69.5% (62.1–76.2)	265/354; 74.9% (70.0–79.3)
Bleb measured		158	154	94/158; 59.5% (51.4–67.2)	65/154; 42.2% (34.3–50.4)	159/312; 51.0% (45.3–56.6)	89/158; 56.3% (48.2–64.2)	65/154; 42.2% (34.3–50.4)	154/312; 49.4% (43.7–55.1)	126/158; 79.7% (72.6–85.7)	105/154; 68.2% (60.2–75.4)	231/312; 74.0% (68.8–78.8)
Bleb size, mm	0	5/158 (3.2%)	19/154 (12.3%)	2/5; 40.0% (5.3–85.3)	8/19; 42.1% (20.3–66.5)	10/24; 41.7% (22.1–63.4)	3/5; 60.0% (14.7–94.7)	6/19; 31.6% (12.6–56.6)	9/24; 37.5% (18.8–59.4)	4/5; 80.0% (28.4–99.5)	9/19; 47.4% (24.4–71.1)	13/24; 54.2% (32.8–74.4)
	≤4	3/158 (1.9%)	15/154 (9.7%)	2/3: 66.7% (9.4–99.2)	5/15; 33.3% (11.8–61.6)	7/18; 38.9% (17.3–64.3)	2/3; 66.7% (9.4–99.2)	4/15; 26.7% (7.8–55.1)	6/18; 33.3% (13.3–59.0)	2/3; 66.7% (9.4–99.2)	11/15; 73.3% (44.9–92.2)	13/18; 72.2% (46.5–90.3)
	5	12/158 (7.6%)	26/154 (16.9%)	4/12; 33.3% (9.9–65.1)	7/26; 26.9% (11.6–47.8)	11/38; 28.9% (15.4–45.9)	7/12; 58.3% (27.7–84.8)	10/26; 38.5% (20.2–59.4)	17/38; 44.7% (28.6–61.7)	9/12; 75.0% (42.8–94.5)	14/26; 53.8% (33.4–73.4)	23/38; 60.5% (43.4–76.0)
	6	12/158 (7.6%)	30/154 (19.5%)	7/12; 58.3% (27.7–84.8)	11/30; 36.7% (19.9–56.1)	18/42; 42.9% (27.7–59.0)	7/12; 58.3% (27.7–84.8)	7/30; 23.3% (9.9–42.3)	14/42; 33.3% (19.6–49.5)	11/12; 91.7% (61.5–99.8)	21/30; 70.0% (50.6–85.3)	32/42; 76.2% (60.5–87.9)
	7	35/158 (22.2%)	41/154 (26.6%)	19/35; 54.3% (36.6–71.2)	19/41; 46.3% (30.7–62.6)	38/76; 50.0% (38.3–61.7)	19/35; 54.3% (36.6–71.2)	22/41; 53.7% (37.4–69.3)	41/76; 53.9% (42.1–65.5)	24/35; 68.6% (50.7–83.1)	28/41; 68.3% (51.9–81.9)	52/76; 68.4% (56.7–78.6)
	8	59/158 (37.3%)	22/154 (14.3%)	35/59; 59.3% (45.7–71.9)	14/22; 63.6% (40.7–82.8)	49/81; 60.5% (49.0–71.2)	32/59; 54.2% (40.8–67.3)	15/22; 68.2% (45.1–86.1)	47/81; 58.0% (46.5–68.9)	48/59; 81.4% (69.1–90.3)	21/22; 95.5% (77.2–99.9)	69/81; 85.2% (75.6–92.1)
	9	22/158 (13.9%)	1/154 (0.7%)	16/22; 72.7% (49.8–89.3)	1/1; 100.0% (2.5–100.0)	17/23; 73.9% (51.6–89.8)	13/22; 59.1% (36.4–79.3)	1/1; 100.0% (2.5–100)	14/23; 60.9% (38.5–80.3)	19/22; 86.4% (65.1–97.1)	1/1; 100.0% (2.5–100)	20/23; 87.0% (66.4–97.2)
	10	10/158 (6.3%)	0/154 (0.0%)	9/10; 90.0% (55.5–97.7)	0/0; …	9/10; 90.0% (55.5–99.7)	6/10; 60.0% (26.2–87.8)	0/0; …	6/10; 60.0% (26.2–87.8)	9/10; 90.0% (55.5–99.7)	0/0; …	9/10; 90.0% (55.5–97.7)
Test for trend^a^		…	…	*P* = .024, *r* = 0.18 (.02–.34)	*P* = .042,*r* = 0.16 (.01–.32)	*P* < .001,*r* = 0.21 (.09–.32)	*P* = .94, *r* = –0.01 (–.16 to .15)	*P* < .001, *r* = 0.27 (.11–.43)	*P* = .002, *r* = 0.18 (.06–.30)	*P* = .126, *r* = 0.12 (–.04 to .28)	*P* = .006, *r* = 0.22 (.06–.38)	*P* = .001, *r* = 0.18 (.07–.29)

Abbreviations: CI, confidence interval; DSJI, disposable syringe jet injector; ID, intradermal; N&S, needle and syringe.

^a^Spearman rank correlation coefficient (*r*) (95% confidence intervals) and associated significance level.

Of the 100-µL fractional dose volume, the median fluid loss with the N&S was between 2.5 µL and 5.0 µL, although >40.0 µL was lost in 6.3% of injections. The median fluid loss with the DSJI was between 5.0 μL and 10.0 µL, and 12.6% of injections resulted in >40 µL fluid loss. There was a significant inverse correlation between the volume of vaccine lost onto the skin at the time of injection and the size of the ID bleb generated (*P* = .001). This was maintained when data for N&S-based (*P* = .03), but not DSJI-based, ID fIPV administration were examined separately. The correlation coefficients were low in all cases, suggesting that, particularly in the case of the DSJI, vaccine injected deep to the dermis was also in part responsible for the smaller bleb sizes and hence immune responses reported in some cases (Supplementary Table 5).

There was a significant reduction in serotype 2 immunogenicity associated with increasing volumes of fluid lost onto the skin when the data for all ID vaccinations were analyzed (Supplementary Table 6). The volume of fluid loss had no effect on the immunogenicity IM IPV (data not shown).

## DISCUSSION

This study demonstrates, to our knowledge for the first time, a significant positive correlation between the size of the ID fluid bleb generated at the time of ID fIPV administration and the subsequent immune response. The consistently lower immunogenicity of ID fIPV compared with a full IM dose of the same vaccine reported in previous studies (Supplementary Table 1) was largely overcome when a bleb size of 8–10 mm was generated. The findings are consistent with the one study that has examined the phenomenon previously, which reported a trend in the same direction but was inconclusive [9]. It should be noted that the trial was undertaken in tOPV-primed 9- and 10-month-old infants and this should be considered in applying the findings, although there is little reason to think that the trend reported would be restricted to this population.

The finding has important implications. First, it suggests that when training the large number of vaccinators required, based on recent WHO recommendations, to deliver ID fIPV in campaigns and routinely [5], the focus should be placed on maximizing the bleb size generated. Even a modest increase in the response rate in the population will reduce the compromise inherent in ID fIPV use. Second, the finding should guide the development of the next generation of needle-free and other devices designed to facilitate ID injections. Given the correlation, device optimization based in bleb measurement has the potential to reduce empirical modification and human testing. Finally, the finding may also have broader implications for the future delivery of other vaccines by the ID or cutaneous route, which warrant further assessment.

## Supplementary Data

Supplementary materials are available at *Clinical Infectious Diseases* online. Consisting of data provided by the authors to benefit the reader, the posted materials are not copyedited and are the sole responsibility of the authors, so questions or comments should be addressed to the corresponding author.

## Supplementary Material

Supplementary Data

## Notes

***Acknowledgments.*** We thank Dr Kevin Brown and the staff of the Immunization and Diagnostic and Enteric Virus Units within the Virus Reference Department, Public Health England (Colindale, UK) for conducting the serological testing; the Nederlands Vaccin Instituut for the donation of the IPV vaccine; PharmaJet, which was contracted to supply the DSJI devices and to provide certified training; Laura Saganic, Gene Saxon, and Darin Zehrung from PATH for their contribution to the device evaluation methodology; the government of The Gambia, Ministry of Health and Social Welfare, and Expanded Programme on Immunization office and regional health teams for their support; and all other MRC staff who assisted with the study in any way and the infants and families who took part in the study.

***Financial support.*** This work was funded by a grant from the Bill & Melinda Gates Foundation (grant number OPP1080506) and by the Medical Research Council (UK).

***Potential conflicts of interest.*** A. S. B. is an employee of the Bill & Melinda Gates Foundation, which provided grant funding for the trial. R. C. has received grants from the Bill & Melinda Gates Foundation during the conduct of this trial. B. K. has previously received grant funding from GSK and Pfizer to conduct vaccine research although not for vaccines in any way related to those used in this trial. M. R. was Chief Science Officer for PharmaJet during the performance of this study, and is now CEO/President of Thrivant Health, Inc. All other authors report no potential conflicts. All authors have submitted the ICMJE Form for Disclosure of Potential Conflicts of Interest. Conflicts that the editors consider relevant to the content of the manuscript have been disclosed.
