# Supplementary material for: The Immunogenicity of Fractional Intradermal Doses of the Inactivated Poliovirus Vaccine Is Associated With the Size of the Intradermal Fluid Bleb
Source: Clin Infect Dis. 2017 Apr 21;65(5):851–4. doi: 10.1093/cid/cix381 (PMC5850454; doi:10.1093/cid/cix381)
Supplement: Supplementary Data [file cix381_suppl_supplementary_data.pdf]

Table S1: A summary of previous trials of fractional dose IPV administration.

Country	Population	Trial/study design	ID fIPV	Comparator	Serological endpoints	Outcomes	Other serological/ immune endpoints	Ref
India<br>1991	10adults<br>8 children.<br>All seropositive	No comparator	1 x 0.1mL ID N&S	-	GMT	Post-vaccination GMT<br>Adults vs. Children<br>ST1: 1328 vs. 588<br>ST2: 861 vs. 446<br>ST3: 1448 vs. 630	No association between GMT and local reaction size.	[1]
India<br>1992	OPV and IPV naive 6 week old infants<br>n=9 [available for analysis]	Non-randomized<br>Historical IM comparator data	2 x 0.1mL<br>ID N&S<br>8 week interval<br>6 and 14 weeks	2 x 0.5mL<br>IM N&S<br>8 week interval<br>6 and 14 weeks	Seroconversion [seronegative to seropositive or 4 fold titre rise based on calculated maternal antibody decay]<br>Seroconversion index - mean seroconversion across all 3 poliovirus serotypes<br>4 weeks after second vaccination	Seroconversion ID<br>ST1: 78%<br>ST2: 89%<br>ST3: 78%<br>Seroconversion index ID - 82%<br>Seroconversion index IM - 91%	-	[2]
India<br>1998	OPV and IPV naive 6 to 8 week olds<br>n=69 [available for analysis]	Randomized	2 x 0.1mL<br>ID N&S<br>8 week interval<br>6 and 14 weeks<br>(n=30)	3 x 0.1mL<br>ID N&S<br>4 week interval<br>6, 10 and 14 weeks<br>(n=39)	Seroconversion [seronegative to seropositive or 4 fold titre rise based on calculated maternal antibody decay]<br>Seroconversion index - mean seroconversion across all 3 poliovirus serotypes<br>4 weeks after second vaccination	Seroconversion (GMT)<br>2 doses ID vs. 3 doses ID<br>ST1: 90% (330) vs. 90% (120)<br>ST2: 70% (223) vs. 80% (125)<br>ST3: 97% (133) vs. 98% (90)<br>Seroconversion index:<br>85.5% vs. 89.0%<br>All non-significant at p=0.05	Reduced seroconversion rates associated with the presence of maternal antibodies	[3]
Oman<br>2010	OPV and IPV naive 2 month olds<br>n=373 [available for analysis]	Randomized	3 x 0.1mL<br>ID Biojector 2000, Bioject<br>2 month interval<br>2, 4 and 6 months<br>(n=187)	3 x 0.5mL<br>IM N&S<br>2 month interval<br>2, 4 and 6 months<br>(n=186)	Seroconversion<br>[4 fold titre rise based on calculated maternal antibody decay]	Seroconversion (GMT)<br>ID vs. IM<br>ST 1: 97.3% (228) vs. 100.0% (724)<br>ST 2: 95.7% (287) vs. 100.0% (1149)*<br>ST 3 :97.9% (362) vs. 100.0% (≥1448)<br>*p<0.01 for seroconversion<br>All titres p<0.001	ST1 poliovirus excretion on day 7 post mOPV1 challenge<br>63.1% IM<br>74.8 % ID<br>P=0.03	[4]

Country	Population	Trial/study design	ID fIPV	Comparator	Serological endpoints	Outcomes	Other endpoints	Ref
Cuba<br>2010	OPV and IPV naive 6 week olds<br>n=364<br>[available for analysis]	Randomized	3 x 0.1mL<br>ID Biojector 2000, Bioject<br>4 week interval<br>6, 10 and 14 weeks<br>(n=187)	3 x 0.5mL<br>IM N&S<br>4 week interval<br>6, 10 and 14 weeks<br>(n=177)	Seroconversion [seronegative to seropositive or 4 fold titre rise based on calculated maternal antibody decay]<br>Reciprocal median antibody titres	Seroconversion (Reciprocal median antibody titres)<br>ID vs. IM<br>ST 1: 52.9% (19) vs. 89.3% (85)<br>ST 2: 85.0% (45) vs. 95.5% (214)<br>ST 3: 69.0% (32) vs. 98.9% (295)<br>$p \leq 0.001$ for all comparisons	Strong association between maternal antibody levels (higher level quartile) and the failure of infants to seroconvert.	[5]
Philippines<br>2011	OPV and IPV naive 6 week olds<br>n=223 [available for analysis]	Randomized<br>4 week intervals<br>6, 10 and 14 weeks. Booster dose at 15-18 months	1 x 0.1mL<br>ID N&S<br>(n=115)	1 x 0.5mL<br>IM N&S<br>(n=115)	Seroprotection<br>Seroconversion [seronegative to seropositive or 4 fold titre rise based on calculated maternal antibody decay]<br>GMT	ID vs. IM - post-primary series<br>Seroprotection (%) (GMT)<br>ST1: 100.0% (221) vs. 100.0% (585)<br>ST2: 100.0% (234) vs. 100.0% (795)<br>ST3: 99.1% (194) vs. 100.0% (774)<br>ID non-inferior to IM for all serotypes<br>Seroconversion (%)<br>ST1: 99.1 vs. 98.2<br>ST2: 94.5 vs. 98.2<br>ST3: 95.4 vs. 100.0	ID vs. IM - post-booster<br>Seroconversion (%)<br>ST1: 95.5 vs. 96.4<br>ST2: 83.8 vs. 88.3<br>ST3: 94.6 vs. 94.6	[6]
India<br>2012	tOPV and mOPV2 primed 6 to 9 month old infants<br>n=869 [available for analysis]	Randomized<br>Single dose at 6 to 9 months	1 x 0.1mL<br>GSK IPV<br>ID Tropis, Pharmajet<br>(n=168)	1 x 0.5mL<br>IM N&S<br>[GSK (n=183) and Panacea (n=169) IPV compared]<br>mOPV1 standard potency<br>(n=175)<br>mOPV1 high potency<br>(n=174)	Seroconversion [seronegative to seropositive]<br>Increase in titres [4 fold rise in infants seropositive at baseline]<br>Reciprocal median antibody titres	ID vs. IM [GSK vaccine]<br>Seroconversion<br>ST1: 100% vs. n/a<br>ST2: 59% vs. 100%†<br>ST3: 36% vs. 90%†<br>†p<0.0001<br>Increase in titres<br>ST1: 56% vs. 86%‡<br>ST2: 43% vs. 70%*<br>ST3: 68% vs. 90%‡<br>‡p<0.05; *p<0.01<br>Reciprocal median antibody titres<br>ST1: ≥ 1448 vs. ≥ 1448<br>ST2: 724 vs. ≥ 1448†<br>ST3: 202 vs. 455†<br>†p<0.0001	Immunogenicity of IM N&S Panacea IPV and mOPV1 [standard and high potency] reported.	[7]

Country	Population	Trial/study design	ID fIPV	Comparator	Serological endpoints	Outcomes	Other endpoints	Ref
Cuba<br>2013	OPV and IPV naive 4 month olds<br>n=310<br>[available for analysis]	Randomized<br>4 and 8 month doses	2 x 0.1mL<br>ID Biojector 2000, Bioject<br>(n=157)	2 x 0.1mL<br>IM N&S<br>(n=153)	Seroconversion [seronegative to seropositive or 4 fold rise in infants seropositive at baseline]<br>Reciprocal median antibody titres	ID vs. IM<br>Seroconversion (%) (Reciprocal median antibody titres)<br>Post-first (4 month) dose:<br>ST1: 16.6% (<8) vs. 46.4% (11)‡<br>ST2: 47.1% (9) vs. 62.7% (28)*<br>ST3: 14.6% (<8) vs. 32.0% (<8)‡<br>‡p<0.001; p=0.008<br>Post-second (8 month) dose (cumulative over 2 doses):<br>ST1: 93.6% (450) vs. 100.0% (≥1448)†<br>ST2: 98.1% (898) vs. 100.0% (≥ 1448)<br>ST3: 93.0% (71) vs. 99.3% (898)¶<br>†p=0.002; ¶p=0.006	Priming response<br>[seronegative to seropositive or 4 fold rise in infants seropositive between 8 month and 8 months 7 days]<br>ID vs. IM<br>ST1: 90.8 vs. 97.6<br>ST2: 94.0 vs. 98.2<br>ST3: 89.6 vs. 98.1 (p<0.01)	[8]
Netherlands<br>2013	IPV primed adults<br>n=125 [available for analysis]	Randomized<br>Single dose	1 x 0.1mL<br>ID DSJI, Pharmajet<br>(n=32)	1 x 0.5mL<br>IM N&S<br>(n=32)<br>IM DSJI, Pharmajet<br>(n=30)<br>1 x 0.1mL<br>IM N&S<br>(n=31)	GMT ($\log_2$)	ID vs. IM N&S<br>ST1: 6.94 vs. 7.14<br>ST2: 7.71 vs. 8.13<br>ST3: 6.19 vs. 7.26<br>Non-inferiority not demonstrated for ID N&S vs. IM N&S	Non-inferiority not demonstrated for IM DSJI (0.5mL) or IM N&S (0.1mL) compared to IM N&S (0.5mL)	[9]
Bangladesh<br>2015	OPV and IPV naive 6 week olds<br>n=922<br>[available for analysis]	Randomized<br>6 (10) and 14 week doses	2 x 0.1mL ID<br>NanoPass MicronJet 600<br>(n=152)	2 x 0.5mL<br>IM N&S<br>(n=156)<br>3 x tOPV<br>(n=203)<br>3 x bOPV<br>(n=200)<br>2 x 0.1mL ID + 1 x bOPV<br>(n=211)	Seroconversion [seronegative to seropositive or 4 fold rise in infants seropositive at baseline after adjusting for the decay in maternal antibodies]	ID vs. IM N&S<br>Seroconversion (%)<br>ST1: 87.5% vs. 94.9%<br>ST2: 80.9% vs. 91.0%<br>ST3: 88.8% vs. 97.4%<br>Non-inferiority not demonstrated for ID vs. IM routes.	ID vs. IM N&S<br>Poliovirus shedding 1 week after tOPV challenge at 18 weeks<br>ST1: 48.3% vs. 49.4%<br>ST2: 65.5% vs. 57.1%<br>ST3: 42.4% vs. 32.1%<br>Non-significant	[10]

Country	Population	Trial/study design	ID fIPV	Comparator	Serological endpoints	Outcomes	Other endpoints	Ref
Cuba<br>2015	OPV primed<br>12 to 20 months of age<br>n=728 [available for analysis]	Randomized<br>Single dose	1 x 0.1mL<br>(A) ID N&S<br>(n=134)<br>(B) ID Biojector 2000, Bioject<br>(n=145)<br>(C) ID pen, Bioject<br>(n=153)<br>(D) ID DSJI, Pharmajet<br>(n=150)	1 x 0.5mL<br>IM N&S<br>(n=146)	Immune response<br>[seronegative to seropositive or 4 fold rise in infants seropositive at baseline]<br>Reciprocal median antibody titres	A vs. B vs. C vs. D vs. IM N&S<br>Immune response (%)<br>ST1: 48.8% vs. 59.3% vs. 40.4% vs. 53.8% vs. 89.5%<br>ST2: 49.4% vs. 51.7% vs. 22.6% vs. 54.5% vs. 87.9%<br>ST3: 78.8% vs. 82.4% vs. 44.6% vs. 74.2% vs. 96.8%<br>Reciprocal median antibody titres<br>ST1: 1423 vs. 1423 vs. 898 vs. 1423 vs. 4499<br>ST2: 1130 vs. 1130 vs. 566 vs. 1130 vs. 2839<br>ST3: 1130 vs. 1423 vs. 566 vs. 1423 vs. 4499	Immune response by ID administration method affected vaccine loss for ST2. No significant effect of bleb measurement	[11, 12]
United States<br>2015	Adults with well controlled HIV infections<br>OPV +/- IPV primed according to age based on US schedule<br>n=224 [available for analysis]	Randomized<br>Single dose	(A) 0.2mL x 1<br>(n=65)<br>(B) 0.1mL x 1<br>(n=63)<br>ID NanoPass MicronJet 600	(C) 0.5mL x 1<br>(n=64)<br>(D) 0.2mL x 1<br>(n=32)<br>IM N&S	Immune response<br>[seronegative to seropositive or 4 fold rise in infants seropositive at baseline]<br>Geometric mean titres (GMT)	(A) vs. (B) vs. (C) vs. (D)<br>Immune response (%)<br>ST1: 91% vs. 84% vs. 92% vs. 94%<br>ST2: 92% vs. 84% vs. 94% vs. 94%<br>ST3: 91% vs. 87% vs. 98% vs. 97%<br>All non-significant except ST2 p=0.01 (B) vs.(C)<br>GMT<br>ST1: 1715 vs. 976 vs. 1249 vs. 1328<br>ST2: 2188 vs. 1438. vs. 1489 vs. 1938<br>ST3: 2375 vs. 1698 vs. 1792. vs. 2075<br>All non-significant		[13]

Country	Population	Trial/study design	ID fIPV	Comparator	Serological endpoints	Outcomes	Other endpoints	Ref
The Gambia<br>2016	OPV primed 9 and 10 month olds<br>n=754	Randomized<br>Single dose	0.1mL x 1<br>(A) ID N&S<br>(B) ID Tropis, Pharmajet	0.5mL x 1<br>(C) IM N&S<br>(D) IM Stratis Pharmajet	Immune response<br>[seronegative to seropositive or 4 fold rise in infants seropositive at baseline]<br>Reciprocal median antibody titres	(A) vs. (B) vs. (C) vs. (D)<br>Immune response (%)<br>ST1: 55.9 vs. 43.5 vs. 66.9 vs. 66.1<br>ST2: 58.2 vs. 41.8 vs. 72.5 vs. 59.5.<br>ST3: 80.2 vs. 69.5 vs. 82.6 vs. 81.0.<br>Non inferiority not shown for IM N&S vs. either ID route<br>Reciprocal median antibody titres<br>ST1: 256 vs. 256 vs. 512 vs. 512<br>ST2: 256 vs. 256 vs. 512 vs. 512<br>ST3: 256 vs. 256 vs. 1024 vs. 512<br>Non inferiority not shown for IM N&S vs. either ID route		[14]

GMT – geometric mean antibody titres; OPV – oral poliovirus vaccine; ID – intradermal; IM – intramuscular; N&S – needle and syringe; ST - serotype

Table S2: Inclusion and Exclusion Criteria

**Inclusion Criteria**
• Nine to ten months of age inclusive<br>• Receipt of at least three doses of tOPV (excluding a dose given at birth)<br>• Informed consent for trial participation obtained from a parent (or guardian only if neither parent is alive or if guardianship has been legally transferred)<br>• Resident in the study area and with no plans to travel outside the study area during the period of subject participation<br>• Willingness and capacity to comply with the study protocol as judged by a member of the clinical trial team
**Exclusion Criteria**
• Use of any IMP within the 28 days preceding enrolment<br>• Receipt of OPV (any valency) with the 28 days preceding enrolment<br>• Planned administration of any vaccine outside those defined in the study protocol at anytime during trial participation<br>• Previous receipt of a measles, rubella, yellow fever or IPV vaccine<br>• BCG vaccination in the month prior to recruitment<br>• Any suspected or confirmed congenital or acquired state of immune deficiency including but not limited to primary immunodeficiencies including thymus disorders, HIV/AIDS, hematological or lymphoid malignancies (blood tests will <u>not</u> be routinely undertaken with this regard as part of the study[1])<br>• Any current immunosuppressive/immunomodulatory medication or treatment including, but not limited to corticosteroids, cyclosporin, azathioprine, cyclophosphamide, methotrexate, radiotherapy, bone marrow transplantation<br>• Receipt of any immunosuppressive or immunomodulatory medication or treatment within the six months preceding trial enrolment (for corticosteroids this is defined as a dose of prednisolone (or equivalent) of greater than 2mg/kg/day for one week or 1mg/kg/day for one month. The use of inhaled or topical corticosteroids is not an exclusion criteria<br>• Receipt of pooled human immunoglobulin, other blood product or any monoclonal antibody therapy at any point prior to recruitment or plans to receive such therapy at any point during the trial<br>• Any significant congenital defect or significant chronic health problem (e.g. chronic hematological (including severe anemia), renal, gastrointestinal, respiratory, neurological and cardiovascular disorders).<br>• A history of anaphylactic or anaphylactoid reaction to egg, chicken proteins, neomycin, streptomycin polymyxin B, any previous vaccination or any individual component of one of the vaccines<br>• Confirmed fructose intolerance<br>• Severe protein-energy malnutrition (z-score of less than -3)<br>• Any clinically suspected or confirmed congenital or acquired clotting or bleeding disorder or any mediation known to significantly interfere with clotting (e.g. hemophilia or current anti-coagulant therapy) (blood tests will <u>not</u> be routinely undertaken with this regard as part of the study)<br>• Any other condition which, in the opinion of the sub-investigator or ultimately the PI, is likely to interfere with the assessment of the primary or exploratory objectives<br>• Any significant signs or symptoms of an acute illness or infection including an axillary temperature >38.0°C or <u>documented</u> fever >38°C in the preceding 48 hours<br>If the reason for ineligibility is likely to be temporary (e.g. a fever >38°C, receipt of OPV within the preceding 28 days etc) and either will or may resolve before the infant reaches 11 months they will not be recorded as a screening failure but instead will be re-screened within an appropriate future time-window (e.g. at least 48 hours after the last documented fever of >38°C or at least 28 days after the receipt of OPV) and a decision made regarding eligibility at that point.

[1]The prevalence of HIV identified during antenatal screening in this part of The Gambia is around 2 percent

Table S3: Pre- and post-vaccination poliovirus seropositivity and immune responses

	IM N&S	IM DSJI	ID N&S	ID DSJI
	n = 178	n = 168	n = 177	n = 177
**Serotype 1**				
Pre-vaccination seropositivity % (95% CI)	87.1% (155/178) (81.4 to 91.2)	89.9% (151/168) (84.4 to 93.6)	85.9% (152/177) (80.0 to 90.2)	89.8% (159/177) (84.5 to 93.5)
Total response % (95% CI)	66.9%(119/178) (59.6 to 73.3)	66.1%(111/168) (58.6 to 72.8)	55.9%(99/177) (48.6 to 63.0)	43.5%(77/177) (36.4 to 50.9)
Post-vaccination seropositivity % (95% CI)	98.3% (175/178) (95.2 to 99.4)	99.4% (167/168) (96.7 to 99.9)	98.3% (174/177) (95.1 to 99.4)	97.2% (172/177) (93.6 to 98.8)
**Serotype 2**				
Pre-vaccination seropositivity % (95% CI)	96.1% (171/178) (92.1 to 98.1)	98.8% (166/168) (95.8 to 99.7)	97.7% (173/177) (94.3 to 99.1)	98.9% (175/177) (96.0 to 99.7)
Total response % (95% CI)	72.5%(129/178) (65.5 to 78.5)	59.5%(100/168) (52.0 to 66.7)	58.2%(103/177) (50.8 to 65.2)	41.8%(74/177) (34.8 to 49.2)
Post-vaccination seropositivity % (95% CI)	99.4% (177/178) (96.9 to 99.9)	100.0% (168/168) (97.8 to 100.0)	98.9% (175/177) (96.0 to 99.7)	100.0% (177/177) (97.9 to 100.0)
**Serotype 3**				
Pre-vaccination seropositivity % (95% CI)	96.1% (171/178) (92.1 to 98.1)	98.8% (166/168) (95.8 to 99.7)	97.7% (173/177) (94.3 to 99.1)	98.9% (175/177) (96.0 to 99.7)
Total response % (95% CI)	82.6%(147/178) (76.3 to 87.5)	81.0%(136/168) (74.3 to 86.2)	80.2%(142/177) (73.7 to 85.4)	69.5%(123/177) (62.4 to 75.8)
Post-vaccination seropositivity % (95% CI)	98.3% (175/178) (95.2 to 99.4)	96.4% (162/168) (92.4 to 98.4)	94.4% (167/177) (89.9 to 96.9)	92.7% (164/177) (87.8 to 95.7)

Data are percentages (n/N) plus 95% confidence intervals. Seropositivity is defined as the percentage of infants with a serotype-specific poliovirus neutralizing antibody titre of ≥8 (pre-vaccination or four to six weeks post-vaccination. Total response includes the percentage of infant who were seronegative (poliovirus neutralizing antibody titre of <8) pre-vaccination and became seropositive (poliovirus neutralizing antibody titre of ≥8) post-vaccination plus the percentage of infants who were seropositive at baseline and then had a four-fold rise in neutralizing antibody titres following vaccination.

Table S4: Association between bleb size and post vaccination antibody titres

	ID N&S n=158		ID DSJI n=154		Combined n=312	
	p value	Spearman correlation coefficient (95% confidence intervals)	p value	Spearman correlation coefficient (95% confidence intervals)	p value	Spearman correlation coefficient (95% confidence intervals)
**Serotype 1**	0.411	0.12 (-0.05 – 0.27)	0.578	0.12 (-0.04 – 0.28)	0.023	0.20 (0.09 – 0.31)
**Serotype 2**	0.312	0.05 (-0.11 – 0.21)	0.007	0.24 (0.08 – 0.40)	<0.001	0.22 (0.11 – 0/33)
**Serotype 3**	0.082	0.17 (0.01 – 0.32)	0.021	0.27 (0.11 – 0.42)	0.001	0.24 (0.13 – 0.36)

Table S5: Association between fluid loss onto the skin at the time of injection and bleb size

		p value	Spearman correlation coefficient (95% confidence intervals)
**ID N&S**	n = 128	0.033	-0.189<br>(-0.364 - -0.013)
**ID DSJI**	n = 132	0.193	-0.114<br>(-0.287 - 0.058)
**Combined data**	n = 260	0.001	-0.207<br>(-0.329 - -0.085)

Table S6: Fluid loss at the time of injection and total responses to fractional intradermal doses of IPV

		Fluid Loss		Serotype 1			Serotype 2			Serotype 3		
				Total response (seroconversion + 4-fold titre rise)			Total response (seroconversion + 4-fold titre rise)			Total response (seroconversion + 4-fold titre rise)		
		n/N (%)		n/N; % (95% CI)			n/N; % (95% CI)			n/N; % (95% CI)		
		ID N&S	ID DSJI	ID N&S	ID DSJI	Combined	ID N&S	ID DSJI	Combined	ID N&S	ID DSJI	Combined
All data		177	177	99/177; 55.9% (48.3 - 63.4)	77/177; 43.5% (36.1 - 51.1)	176/354; 49.7% (44.4 - 55.1)	103/177; 58.2% (50.6 - 65.5)	74/177; 41.8% (34.5 - 49.4)	177/354; 50.0% (44.7 - 55.3)	142/177; 80.2% (73.6 - 85.8)	123/177; 69.5% (62.1 - 76.2)	265/354; 74.9% (70.0 - 79.3)
Fluid loss measured		142	151	80/142; 56.3% (47.8 - 64.6)	67/151; 44.4% (36.3 - 52.7)	147/293; 50.2% (44.3 - 56.0)	81/142; 57.0% (48.5 - 65.3)	62/151; 41.1% (33.1 - 49.3)	143/293; 48.8% (42.9 - 54.7)	114/142; 80.3% (72.8 - 86.5)	107/151; 70.9% (62.9 - 78.0)	221/293; 75.4% (70.1 - 80.2)
Fluid Loss (µL)	0	25/142 (17.6%)	15/151 (9.9%)	11/25; 44.0% (24.4 - 65.1)	9/15; 60.0% (32.3 - 83.7)	20/40; 50.0% (33.8 - 66.2)	15/25; 60.0% (38.7 - 78.9)	11/15; 73.3% (44.9 - 92.2 0	26/40; 65.0% (48.3 - 79.4)	19/25; 76.0% (54.9 - 90.6)	12/15; 80.0% (51.9 - 95.7)	31/40; 77.5% (61.5 - 89.2)
	<2.5	32/142 (22.5%)	41/151 (27.2%)	22/38; 57.9% (40.8 - 73.7)	18/41; 43.9% (28.5 - 60.3)	40/79; 50.6% (39.1 - 62.1)	22/38; 57.9% (40.8 - 73.7)	20/41; 48.8% (32.9 - 64.9)	42/79; 53.2% (41.6 - 64.5)	32/38; 84.2% (68.7 - 94.0)	26/41; 63.4% (46.9 - 77.9)	58/79; 73.4% (62.3 - 82.7)
	2.5 - <5.0	17/142 (12.0%)	13/151 (8.6%)	11/17; 64.7% (38.3 - 85.8)	6/13; 46.2% (19.2 - 74.9)	17/30; 56.7% (37.4 - 74.5)	10/17; 58.8% (32.9 - 81.6)	3/13; 23.1% (5.0 - 53.8)	13/30; 43.3% (25.5 - 62.6)	13/17; 76.5% (50.1 - 93.2)	9/13; 69.2% (38.6 - 90.9)	22/30; 73.3% (54.1 - 87.7)
	5.0 - <10.0	17/142 (12.0%)	22/151 (14.6%)	12/17; 70.6% (44.0 - 89.7)	8/22; 36.4% (17.2 - 59.3)	20/39; 51.3% (34.8 - 67.6)	7/17; 41.2% (18.4 - 67.1)	9/22; 40.9% (20.7 - 63.6)	16/39; 41.0% (25.6 - 57.9)	14/17; 82.4% (56.6 - 96.2)	15/22; 68.2% (45.1 - 86.1)	29/39; 74.4% (57.9 - 87.0)
	10.0 - <20.0	19/142 (13.4%)	20/151 (13.2%)	12/19; 63.2% (38.4 - 83.7)	10/20; 50.0% (27.2 - 72.8)	22/39; 56.4% (39.6 - 72.2)	14/19; 73.7% (48.8 - 90.9)	9/20; 45.0% (32.1 - 68.5 0	23/39; 59.0% (42.1 - 74.4)	17/19; 89.5% (66.9 - 98.7)	15/20; 75.0% (50.9 - 91.3)	32/39; 82.1% (66.5 - 92.5)
	20.0 - ≤ 40.0	17/142 (12.0%)	21/151 (13.9%)	9/17; 52.9% (27.8 - 77.0)	11/21; 52.4% (29.8 - 74.3)	20/38; 52.6% (35.8 - 69.0)	10/17; 58.8% (32.9 - 81.6)	6/21; 28.6% (11.3 - 52.2)	16/38; 42.1% (26.3 - 59.2)	15/17; 88.2% (63.6 - 98.5 0	17/21; 81.0% (58.1 - 94.6)	32/38; 84.2% (68.7 - 94.0)
	>40.0	9/142 (6.3%)	19/151 (12.6%)	3/9; 33.3% (7.5 - 70.1)	5/19; 26.3% (9.1 - 51.2)	8/28; 28.6% (13.2 - 48.7)	3/9; 33.3% (7.5 - 70.1)	4/19; 21.1% (6.1 - 45.6)	7/28; 25.0% (10.7 - 44.9)	4/9; 44.4% (13.7 - 78.8)	13/19; 68.4% (43.4 - 87.4)	17/28; 60.7% (40.6 - 78.5)
Test for Trend				0.722	0.281	0.408	0.637	0.003	0.005	0.786	0.626	0.927

Total response combines all infants who underwent seroconversion and all infants who had a four-fold rise in antibody titres between the pre-vaccination sample and the sample taken four to six weeks post-vaccination. Seroconversion includes all infants who changed from seronegative at baseline to seropositive post-vaccination. Four-fold rise includes all infants who were seropositive at baseline and had a four-fold rise in antibody titres post-vaccination. Seronegative is defined as a reciprocal poliovirus neutralizing antibody titres of <8. Seropositive is defined as a reciprocal poliovirus neutralizing antibody titres of ≥8. Test for trend shows a p-value for increasing fluid loss between responders and non-responders in either group, or in the combined results; ID N&S - intradermal needle and syringe; ID DSJI - intradermal disposable-syringe jet-injector (Tropis™)

1. Samuel BU, Cherian T, Sridharan G, Mukundan P, John TJ. Immune response to intradermally injected inactivated poliovirus vaccine. Lancet 1991; 338(8763): 343-4.
2. Samuel BU, Cherian T, Rajasingh J, Raghupathy P, John TJ. Immune response of infants to inactivated poliovirus vaccine injected intradermally. Vaccine 1992; 10(2): 135.
3. Nirmal S, Cherian T, Samuel BU, Rajasingh J, Raghupathy P, John TJ. Immune response of infants to fractional doses of intradermally administered inactivated poliovirus vaccine. Vaccine 1998; 16(9-10): 928-31.
4. Mohammed AJ, AlAwaidy S, Bawikar S, et al. Fractional doses of inactivated poliovirus vaccine in Oman. N Engl J Med 2010; 362(25): 2351-9.
5. Resik S, Tejeda A, Lago PM, et al. Randomized controlled clinical trial of fractional doses of inactivated poliovirus vaccine administered intradermally by needle-free device in Cuba. J Infect Dis 2010; 201(9): 1344-52.
6. Cadorna-Carlos J, Vidor E, Bonnet MC. Randomized controlled study of fractional doses of inactivated poliovirus vaccine administered intradermally with a needle in the Philippines. Int J Infect Dis 2012; 16(2): e110-6.
7. Estivariz CF, Jafari H, Sutter RW, et al. Immunogenicity of supplemental doses of poliovirus vaccine for children aged 6-9 months in Moradabad, India: a community-based, randomised controlled trial. Lancet Infect Dis 2012; 12(2): 128-35.
8. Resik S, Tejeda A, Sutter RW, et al. Priming after a fractional dose of inactivated poliovirus vaccine. N Engl J Med 2013; 368(5): 416-24.
9. Soonawala D, Verdijk P, Wijmenga-Monsuur AJ, et al. Intradermal fractional booster dose of inactivated poliomyelitis vaccine with a jet injector in healthy adults. Vaccine 2013.

10. Anand A, Zaman K, Estivariz CF, et al. Early priming with inactivated poliovirus vaccine (IPV) and intradermal fractional dose IPV administered by a microneedle device: A randomized controlled trial. Vaccine 2015; 33(48): 6816-22.
11. Resik S, Tejeda A, Mach O, et al. Needle-free jet injector intradermal delivery of fractional dose inactivated poliovirus vaccine: Association between injection quality and immunogenicity. Vaccine 2015; 33(43): 5873-7.
12. Resik S, Tejeda A, Mach O, et al. Immune responses after fractional doses of inactivated poliovirus vaccine using newly developed intradermal jet injectors: a randomized controlled trial in Cuba. Vaccine 2015; 33(2): 307-13.
13. Troy SB, Kouiavskaia D, Siik J, et al. Comparison of the Immunogenicity of Various Booster Doses of Inactivated Polio Vaccine Delivered Intradermally Versus Intramuscularly to HIV-Infected Adults. J Infect Dis 2015; 211(12): 1969-76.
14. Clarke E, Saidu Y, Adetifa JU, et al. Safety and immunogenicity of inactivated poliovirus vaccine when given with measles-rubella combined vaccine and yellow fever vaccine and when given via different administration routes: a phase 4, randomised, non-inferiority trial in The Gambia. The Lancet Global health 2016; 4(8): e534-47.
